# Directable Needle Guide: Efficacy for Image-Guided Percutaneous Interventions

**DOI:** 10.5402/2013/516941

**Published:** 2012-11-27

**Authors:** Hiroshi Ishizaka

**Affiliations:** Department of Radiology, Maebashi Red Cross Hospital, Gunma, Maebashi 371-0014, Japan

## Abstract

Diagnostic and therapeutic image-guided percutaneous interventions have become increasingly important in the clinical management of various conditions. Though precise needle placement via a safe route is essential for successful percutaneous interventions, it is often difficult in cases of deeply situated, small lesions. The present paper describes the efficacy of the directable needle guide (DNG), which allows manipulation of the direction of a fine needle within organs. The DNG was used in patients for needle biopsy of hepatic (*n* = 26) and splenic (*n* = 1) lesions and for percutaneous ethanol injection therapy for liver tumors (*n* = 33) under sonographic or computed tomography guidance. The DNG enabled the direction of a 21- or 22-gauge needle to be successfully changed during needle advancement in all cases, allowing adjustment of the location of the needle tip or needle access root to avoid vessels, the gallbladder, and the lungs. We conclude that DNG increases the safety and ease of percutaneous interventions.

## 1. Introduction

Image-guided percutaneous interventions with a fine needle are used for various clinical purposes, including biopsies, antitumor therapy with ethanol injection, laser thermal ablation, gene-technology implants, and nerve blocks [1–4]. Though precise needle placement passing through a safe root is essential for percutaneous interventions, it is often difficult due to needle deflection and patients' respiratory or postural variations during the procedure and also because of intervening vital structures on the root.

 When a thin beveled needle is inserted into any organ, the tip of the needle has a tendency to curve toward the side when advancing, due to its flexibility. However, when a beveled needle is inserted with a twisting motion, it advances in a straight path. This phenomenon was applied to create a directable needle guide (DNG) that could be used to steer a needle within organs. The present paper describes the utility of the DNG in our clinical experience.

## 2. Subjects and Methods

 The DNG was used in patients undergoing both needle biopsy of hepatic (*n* = 26) or splenic (*n* = 1) lesions and percutaneous ethanol injection therapy for liver tumors (*n* = 33). Sonography and computed tomography (CT) images were used for imaging guidance in 43 and 17 lesions, respectively.

**Figure 1:**
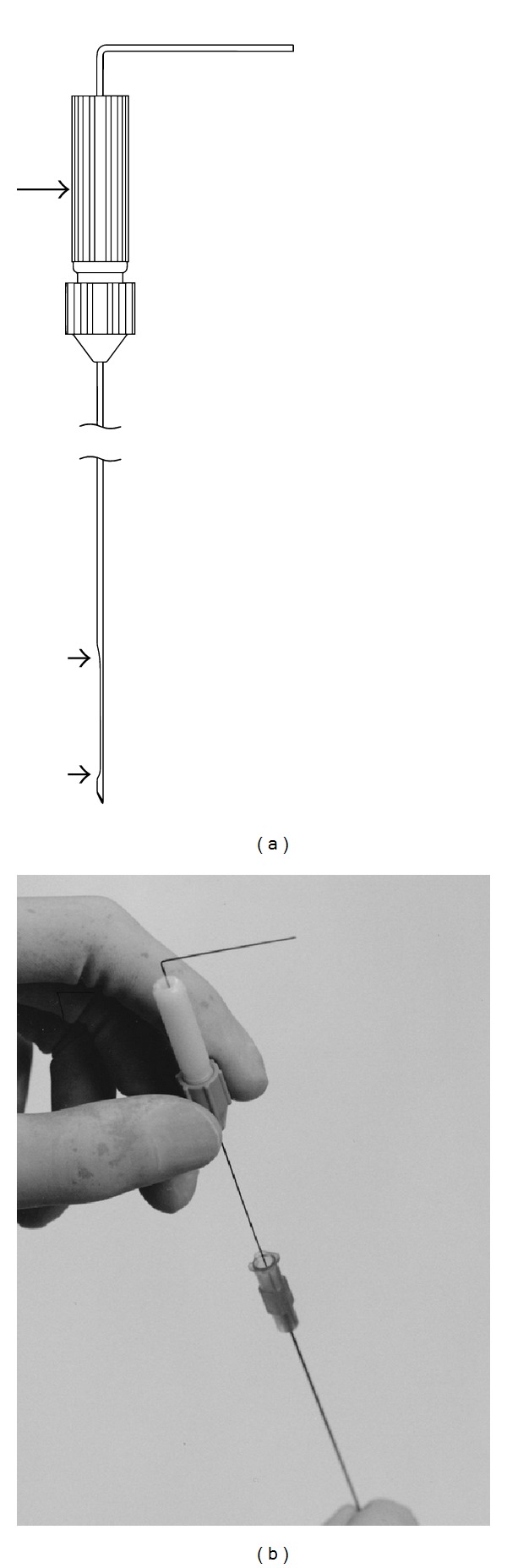
Illustration (a) and photograph (b) of the directable needle guide (DNG). A segmental flattening on the same side as the bevel of the tip (arrowheads) increases the pliancy of the needle in the direction of the bevel side, resulting in curving exclusively to the beveled tip side during advancement within tissues. The tail of the DNG is bent toward the tip side to signify the direction of the curve during advancement. A torque device (arrow) makes it easy to rotate the DNG.

 The DNG (0.018 inches in diameter; 250 mm long) with a flattened segment (0.010 inches in thickness) on the distal portion of the beveled side of the tip (Leadway, Hakko, Tokyo, Japan, a prototype not commercially available at present) (Figures 1(a) and 1(b)) was used to direct the needle tip within the tissue. The DNG was fitted with either a 21- or 22-gauge needle. An end-cutting aspiration biopsy needle (length, 150 mm; Top, Tokyo, Japan) was used for biopsy, and a PTC needle (length, 150 mm; Hakko, Tokyo, Japan) was used for percutaneous ethanol injection therapy.

 After the patient's skin had been cleaned and the entry site anesthetized and incised, a 21- or 22-gauge needle was inserted. In order to change the direction of the needle, the needle stylet was withdrawn, and the DNG was inserted. When the DNG was used in conjunction with CT guidance, the DNG was advanced in a stepwise manner with quick applications of conventional helical 5 mm imaging or interrupted CT-fluoroscopic display to check the DNG tip position. The 21- or 22-gauge needle was then advanced along the DNG.

## 3. Results and Discussion

**Figure 2:**
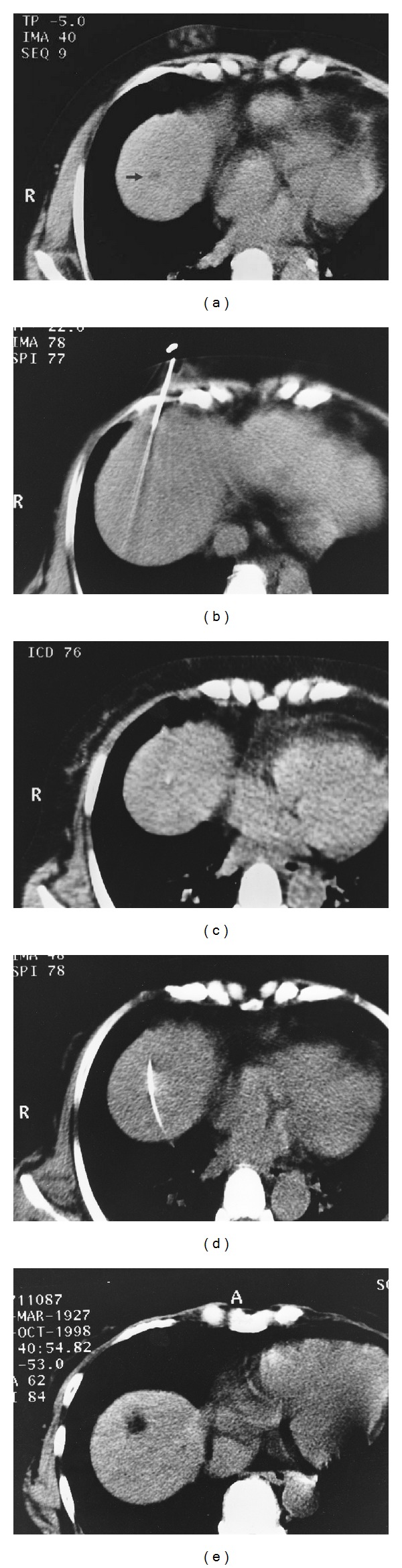
Computed-tomography- (CT-) guided needle approach using a directable needle guide (DNG) for percutaneous ethanol injection. (a) CT image (with tilt of the gantry 33° in the cranial direction) of a 73-year-old woman with hepatitis C shows a hypodense, growing lesion (arrowhead) in the hepatic dome. (b) A 22-gauge injection needle is initially inserted at a position caudal to the CT image plane, avoiding insertion into lung tissue. (c) A DNG (arrow) is advanced in the craniomedial direction to penetrate the lesion under intermittent fluoroscopy. (d) The injection needle is advanced along the DNG. (e) CT image (after replacing the gantry) demonstrates a lesion with decreased attenuation after ethanol injection.

 The DNG was used successfully to steer the needle direction to access the target, and the 21- or 22-gauge needle could be easily advanced along the DNG in all cases. Although the DNG tip was frequently difficult to detect under static conditions on sonography, this could be remedied by moving the DNG back and forth in small motions. The DNG also enabled advancement of the needle while avoiding the hepatic hilar large vessels (*n* = 5), the gallbladder (*n* = 2), and the lungs (*n* = 8) along the needle tracts (Figure 2). When the DNG was used in conjunction with the coaxial biopsy technique (*n* = 9), multiple tissue samples of various portions of the lesion were obtained via the same cannula root by changing the biopsy needle direction.

 The directability of the DNG is provided by a beveled surface of the needle tip and by notable pliancy that is exclusive to the beveled side. The DNG curves intrinsically toward the side of the tip during advancement. However, when the DNG is inserted with a twisting motion, it advances in a straight path. The degree of curve could also be finely controlled, if needed, by manually angling the DNG tip. In this case, the notable pliancy of the DNG tip allowed smooth passage of the DNG through the needle, smooth advancement of the needle along the DNG, and no scratching of the adjacent tissue when the DNG was rotated within the tissue.

Because of the fragility of the flattened segment, the DNG may kink if not used carefully when advancing through hard fibrous tissues. However, in our experience, the DNG did not bend significantly in any direction other than the bevel and did not twist during insertion, even when inserted through tough liver tissue with advanced cirrhosis. Several back-and-forth exploratory motions of the DNG were usually required in order to reach lesions, but these exploratory motions of the extremely fine DNG were far less traumatic when compared with multiple reinsertions of the needle. Although we cannot clarify the extent to which the use of the DNG could reduce the number of additional needle insertions required for repositioning and whether the use of the DNG could reduce the puncture time, we believe that the use of the DNG can enhance the accuracy of puncture and enable intervention for cases that would otherwise be difficult or even impossible with the use of a conventional technique.

## 4. Conclusions

The DNG can finely adjust the needle tract within organs and thereby enables safe, effective, and easy percutaneous intervention for difficult-to-access lesions or in patients who have difficulty holding their breath.
